# Multicenter study of oral and maxillofacial thrombi: analysis of 187 cases

**DOI:** 10.1590/1807-3107bor-2026.vol40.020

**Published:** 2026-03-30

**Authors:** Weslay Rodrigues da SILVA, Caroline Augusta Belo FARIA, Jonathan França da Silva CAVALCANTI, Hannah Gil de Farias MORAIS, Danielle Machado FARIAS, Isadora Vilas Boas CEPEDA, Daniel Augusto Barnabé NOBRE, Victor Zanetti DRUMOND, Bertine Mota Malta Brandão NUNES, Caio César da Silva BARROS, Glória Maria de FRANÇA, Elismauro Francisco de MENDONÇA, Diego Antonio Costa ARANTES, George João Ferreira do NASCIMENTO, Leorik Pereira da SILVA, Cyntia Helena Pereira de CARVALHO, Jefferson da Rocha TENÓRIO, José Alcides Almeida de ARRUDA, Bruno Augusto Benevenuto de ANDRADE, Ana Carolina Uchoa VASCONCELOS, Ana Paula Neutzling GOMES, Lucas Guimarães ABREU, Elaine Judite de Amorim CARVALHO, Danyel Elias da Cruz PEREZ, Ricardo Alves MESQUITA, Hébel Cavalcanti GALVÃO, Roseana de Almeida FREITAS, Ana Paula Veras SOBRAL

**Affiliations:** (a) Universidade de Pernambuco – UPE, School of Dentistry, Department of Oral and Maxillofacial Pathology, Recife, PE, Brazil.; (b) Universidade de São Paulo – USP, School of Dentistry, Department of Stomatology, São Paulo, SP, Brazil.; (c) Universidade Federal do Rio Grande do Norte – UFRN, Oral Pathology and Medicine, Natal, RN, Brazil.; (d) Universidade Federal de Pernambuco – UFPE, Oral Pathology Unit, Department of Clinical and Preventive Dentistry, Recife, PE, Brazil.; (e) Universidade Federal de Pelotas – UFPel, School of Dentistry, Diagnostic Center for Oral Diseases, Pelotas, RS, Brazil.; (f) Universidade Federal de Minas Gerais – UFMG, School of Dentistry, Department of Oral Surgery, Pathology, and Clinical Dentistry, Belo Horizonte, MG, Brazil.; (g) Centro Universitário Cesmac, Master’s Degree in Health Research, Maceió, AL, Brazil.; (h) Universidade Federal de Goiás - UFG, School of Dentistry, Department of Stomatology, Goiânia, GO, Brazil.; (i) Universidade Federal de Campina Grande – UFCG, Oral Histopathology Service, Patos, PB, Brazil.; (j) Universidade Federal do Rio de Janeiro – UFRJ, School of Dentistry, Department of Oral Diagnosis and Pathology, Rio de Janeiro, RJ, Brazil.; (k) Universidade Federal de Minas Gerais – UFMG, School of Dentistry, Department of Child and Adolescent Oral Health, Belo Horizonte, MG, Brazil.

**Keywords:** Diagnosis, Epidemiology, Thrombosis

## Abstract

This study aimed to investigate the clinicopathological characteristics of thrombi in the oral and maxillofacial region. A cross-sectional study was conducted in nine reference centers for oral and maxillofacial pathology diagnosis in Brazil, from which biopsy records were obtained. The data were analyzed descriptively. A total of 93,036 biopsies from the oral and maxillofacial region were evaluated, 187 (0.2%) of which were diagnosed as thrombi. The highest occurrence was in females, with a mean age of 52.4 ± 17.7 years. The lip was the most frequently affected anatomical location. A clinical diagnostic hypothesis of thrombus was considered in 4.2% of cases. Oral thrombi were uncommon lesions in the studied population, with a higher occurrence among females in the seventh decade of life. This study provides a clinicopathological profile of oral and maxillofacial thrombi and offers information that may assist clinicians in establishing a diagnosis.

## Introduction

A thrombus (TO) is characterized by the formation of clots exclusively within blood vessels and/or heart chambers and can lead to partial or total interruption of blood flow.^1-3^. According to Virchow’s triad, TO formation results from alterations in the vascular wall, blood flow, or blood coagulability.^4,5^ The main risk factors for its development include hyperlipidemia, smoking, diabetes, hypertension, obesity, orthopedic surgeries, immobilization, pregnancy, and contraceptive use.^6^

In deep veins, TO can cause ischemia and/or embolism, leading to damage to other organs.^3^ Hence, thrombosis—the result of thrombus formation—is the most common cause of three major cardiovascular diseases: ischemic heart disease, stroke, and venous thromboembolism.^7^ These conditions are more frequent among males older than 65 years.^8,9^ In contrast, superficial vein thrombosis may progress to venous thromboembolism. Deep vein thrombosis of the lower extremities is common and well-studied; however, its occurrence in other, less frequent anatomical locations, such as the oral and maxillofacial region, is poorly understood.^10^ Studies on this region are scarce, and most publications are clinical case reports in which TO is merely described as a morphological finding.

The clinical presentation of oral TO varies. When associated with another lesion, it has an appearance similar to that lesion, representing merely a morphological finding. Conversely, solitary oral thrombi are generally associated with trauma or parafunctional habits and are clinically characterized by increased volume, firm consistency upon palpation, and coloration ranging from purplish to reddish or brownish.^3,11-14^ Furthermore, thrombi in deep sites of the oral and maxillofacial region (i.e., muscles and salivary glands) often exhibit colors similar to those of the mucosa or skin and are detected because of localized swelling.^15,16^ Considering these aspects, this multicenter study aimed to describe the clinicopathological characteristics of thrombi in the oral and maxillofacial region.

## Methods

### Study design and ethics

**Figure 1:**
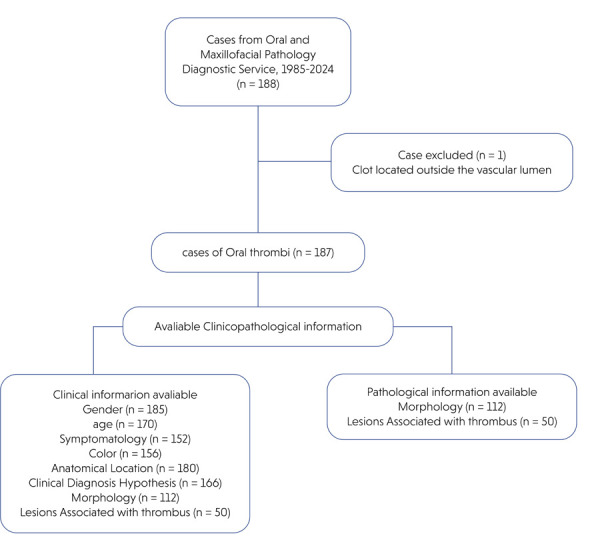
Flowchart illustrating the data collection process.

This cross-sectional observational study was conducted in accordance with the STROBE (Strengthening the Reporting of Observational Studies in Epidemiology) guidelines.^17^ The study was approved by the Research Ethics Committee of Universidade Federal do Rio Grande do Norte (Approval No. 3,728,713) and carried out in accordance with the 1964 Helsinki Declaration and its later amendments. This epidemiological, cross-sectional, clinicopathological multicenter study used secondary data obtained from histopathological examination request forms and reports collected over 39 years (1985–2024). Figure 1 illustrates the selection process. The study population comprised 93,036 cases of oral and maxillofacial biopsies.

Histopathological data were provided by nine oral and maxillofacial pathology diagnostic services from Brazilian universities representing four geographic regions: Universidade Federal do Rio Grande do Norte (Northeast region), Universidade Federal do Campina Grande (Northeast region), Universidade de Pernambuco (Northeast region), Universidade Federal de Pernambuco (Northeast region), Centro Universitário CESMAC (Northeast region), Universidade Federal de Minas Gerais (Southeast region), Universidade Federal do Rio de Janeiro (Southeast region), Universidade Federal de Pelotas (South region), and Universidade Federal de Goiás (Central-West region).

### Sample

The convenience, non_-_probability sampling included all isolated TO cases and thrombi associated with other lesions diagnosed in the oral and maxillofacial region. Data were collected regarding age, symptoms (pain), lesion color, anatomical location, and clinical diagnostic hypotheses. The thrombi were categorized according to their morphological characteristics. The histopathologic diagnoses of the lesions associated with TO cases were also identified and recorded. The sample included cases with available clinical information about the thrombi and sufficient paraffin-embedded specimens for histopathological analysis. Cases involving clots located outside the vascular space were excluded.

### Histopathological analysis

For the histopathological analysis, TO cases were reviewed under conventional light microscopy on glass slides stained with hematoxylin and eosin (HE) by nine oral and maxillofacial pathologists (one from each service), each with more than three decades of experience. TO cases exhibiting clusters of erythrocytes, leukocytes, platelets, and fibrin within the vascular space were considered thrombi.^18,19^Moreover, the TOs were categorized according to their morphological characteristics as follows: a) organizing TO, b) organized TO, b) venous TO, d) mixed TO, and e) unspecified TO.

### Data analysis

The Statistical Package for the Social Sciences (SPSS) software (version 22.0, SPSS, Chicago, USA) was used for data analysis. Descriptive statistics were applied to characterize the cases according to sex, age (grouped by decade of life), symptoms (pain), color, anatomical location, clinical diagnostic hypotheses, morphological characteristics, and presence of thrombus associated with another lesion.

## Results

**Figure 2:**
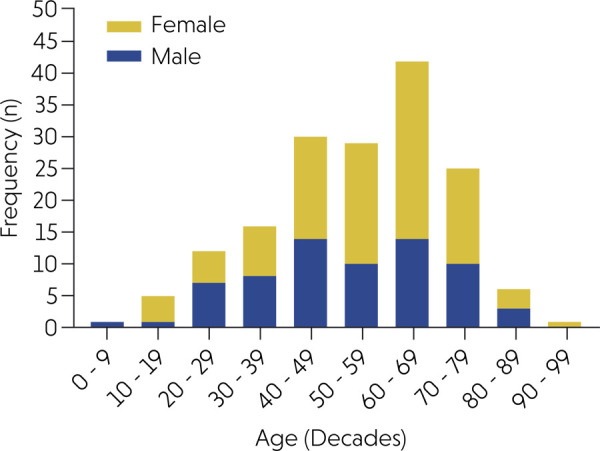
Distribution of 187 cases of oral and maxillofacial thrombi according to Brazilian states and geographic regions (red: Northeast; purple: Southeast; green: Central-West; yellow: South).

A total of 93,036 biopsies from the oral and maxillofacial region were analyzed, 187 (0.20%) of which were diagnosed as TO. One case was excluded after histopathological analysis because it represented a clot located outside the vascular lumen. Table 1 describes the sample characterization in each diagnostic service, and Figure 2 shows the number of oral and maxillofacial thrombus cases in Brazil, according to state and geographic region.

**Table 1:** Characterization of oral and maxillofacial thrombus samples from nine Brazilian reference diagnostic centers.

Centers	State	Years	Total number of biopsies	Number of thrombi (n)	Thrombus frequency (%)
UFRN^a^	Rio Grande do Norte	1985–2024	13,427	31	0.23
UFCG^b^	Paraíba	2016–2024	1,217	3	0.24
UPE^c^	Pernambuco	1990–2024	7,236	14	0.19
UFPE^d^	Pernambuco	1991–2024	8,213	16	0.19
CESMAC^e^	Alagoas	2009–2024	2,103	2	0.01
UFMG^f^	Minas Gerais	1997–2024	12,212	57	0.46
UFRJ^g^	Rio de Janeiro	2012–2024	14,174	17	0.12
UFPeL^h^	Rio Grande do Sul	1991–2024	22,948	43	0.18
UFG^i^	Goiás	2003–2024	11,506	4	0.03
Total	-	-	93,036	187	0.20

^a^Oral Pathology and Medicine, Universidade Federal do Rio Grande do Norte (Northeast region);^b^Oral Histopathology Service, Universidade Federal de Campina Grande (Northeast region);^c^Department of Oral and Maxillofacial Pathology, School of Dentistry, Universidade de Pernambuco (Northeast region); ^d^Oral Pathology Unit, Department of Clinical and Preventive Dentistry, Universidade Federal de Pernambuco (Northeast region);^e^Oral Pathology Laboratory, Centro Universitário CESMAC (Northeast region); ^f^Department of Oral Surgery and Pathology, School of Dentistry, Universidade Federal de Minas Gerais (Southeast region); ^g^Department of Oral Diagnosis and Pathology, School of Dentistry, Universidade Federal do Rio de Janeiro (Southeast region); ^h^Diagnostic Center for Oral Diseases, Universidade Federal de Pelotas (South region); ^i^Department of Stomatology (Oral Pathology), School of Dentistry, Universidade Federal de Goiás (Central-West region).

**Figure 3:**
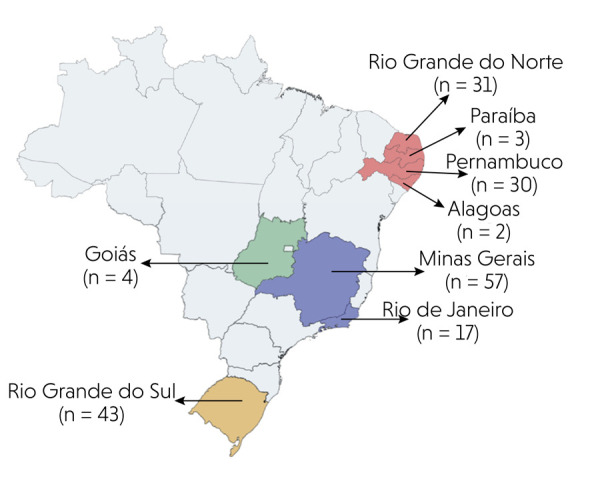
Frequency of oral and maxillofacial thrombi according to age (by decade) and gender (data represented as the number of cases, *n*).

**Figure 4:**
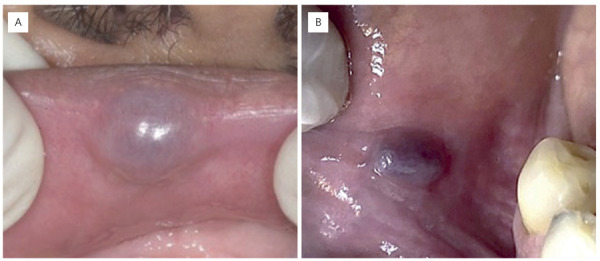
(A) Clinical appearance of an oral thrombus showing a nodular lesion with a smooth surface and purplish color located in the upper lip. (B) Purplish papule on the mucosal surface of the lower lip.

A higher incidence of TO cases was observed among females (56.8%) (female-to-male ratio = 1.3:1). Age ranged from 4 to 90 years, with a mean of 52.4 ± 17.7 years. Age was not reported in 17 cases. A high frequency of cases occurred in the seventh decade of life for both sexes (Figure 3). Regarding symptoms (pain), most lesions were asymptomatic (70%) and exhibited a purplish color (33.7%) (Figure 4A). The most frequently affected anatomical location was the lip (55%), followed by the buccal mucosa (18.2%) and tongue (12.9%) (Table 2). The clinical diagnostic hypothesis of benign vascular lesions—hemangioma, vascular malformation (not otherwise specified), and varix—was indicated in 30.4% of cases, whereas only 4.2% were clinically diagnosed as thrombi.

**Table 2:** Clinicopathological characteristics of the oral and maxillofacial thrombus sample.

Variable	n (%)
Gender (n = 187)	
Female	106 (56.8)
Male	79 (42.2)
Not informed	2 (1)
Symptomatology (n = 187)	
Symptomatic	131 (70)
Asymptomatic	21 (11.2)
Not informed	35 (18.8)
Color (n = 187)	
Purple	63 (33.7)
Blue	26 (13.9)
Brown	24 (12.8)
Red	23 (12.4)
CSTOM	20 (10.7)
Not informed	31 (16.5)
Anatomical location (n = 187)	
Lip	103 (55)
Buccal mucosa	34 (18.2)
Tongue	24 (12.9)
Floor of the mouth	6 (3.2)
Gengiva/alveolar ridge	5 (2.8)
Palate	3 (1.6)
Oral vestibule	2 (1)
Intraosseous	3 (1.6)
Not informed	7 (3.7)
Morphology (n = 187)	
Organizing thrombus	67 (35.9)
Organized thrombus ^*^	22 (11.8)
Venous thrombus	21 (11.2)
Mixed thrombus	2 (1)
Unspecified thrombus	75 (40.1)
Lesions associated with thrombus (n = 50)	
Varix	17 (34)
Vascular malformation NOS	12 (24)
Hemangioma	11 (22)
Actinic Cheilitis	4 (8)
Papillary endothelial hyperplasia	3 (6)
Sialolith	2 (4)
Aneurysmal bone cyst	1 (2)

CSTOM: Color similar to oral mucosa; NOS: Not otherwise specified; * Among the organized thrombi, 4 were channelized and 2 were calcified.

**Figure 5:**
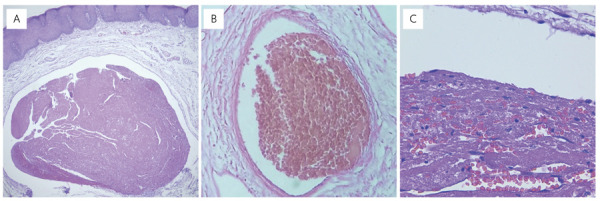
(A) Histopathological findings of oral thrombi. Low-magnification view showing a vascular lumen occluded by a thrombus (hematoxylin and eosin stain). (B) Medium-magnification image showing a thrombus associated with an aneurysmal bone cyst (hematoxylin and eosin stain). (C) High-magnification image showing eosinophilic amorphous material compatible with thrombus; recanalization of the thrombus is also observed (hematoxylin and eosin stain).

In the histopathological analysis, all 187 TO cases exhibited blood vascular spaces containing amorphous and eosinophilic material within the lumen. This material was associated with erythrocyte deposition and inflammatory infiltrate (Figure 5A–C). Most TO cases were morphologically classified as organizing TO (35.9%). Fifty TO cases (26.7%) were associated with another lesion, 34.0% of which were associated with varices (Table 2).

## Discussion

TO have been widely studied because of their clinical impact.^3,20,21^However, in uncommon anatomical locations, such as the oral and maxillofacial region, only few studies have evaluated their incidence, prevalence, or clinicopathological characteristics, including their distribution across affected sites. The scarcity of studies on this subject is probably related to the absence of symptoms and low risk of embolization or mortality in TO of the oral and maxillofacial region.^3,20^ To this end, we investigated the characteristics of thrombi in this region through a multicenter study in a Brazilian population. Accordingly, our results provide important contributions to defining the clinicopathological profile of TO in the oral and maxillofacial region.

In our sample, oral thrombi accounted for 0.2% (n = 187) of all oral and maxillofacial lesions diagnosed. The multicenter analysis revealed a mean patient age of 52.4 years and a slightly higher frequency among females. This mean age is consistent with the findings reported by Tobouti.^20^ Furthermore, our epidemiological profile of thrombi in the oral and maxillofacial region was similar to that reported for other parts of the body.^7,8^ In the context of sex-related risk factors, pregnancy and the use of oral contraceptives are known to increase the risk of thrombus formation, probably because of changes in blood viscosity.^4, 21^ These factors may account for the higher frequency of thrombi among females.

An increase in volume and color change in the affected area were the most common complaints, whereas pain was reported in only 11.2% of cases. In this multicenter study, the lip, buccal mucosa, and tongue were the most frequently affected anatomical sites. These findings are consistent with those reported by Tobouti.^20^ The most frequent clinical diagnostic hypotheses were benign vascular lesions (30.4%); in contrast, only 4.2% of these hypotheses were thrombi. Benign vascular lesions in the oral and maxillofacial region typically exhibit a purplish or reddish color because of erythrocyte accumulation.^22-24^ These lesions should therefore be considered in the differential diagnosis of thrombi. It is also noteworthy that diascopy tends to be negative during clinical examination of oral thrombi cases or may show partial local ischemia.^11,14^ Because the vascular lumen is obstructed by the thrombus, diascopy can yield a negative result. Consequently, thrombi should be considered as clinical diagnostic hypotheses in purple or red vascular lesions that are negative on diascopy. Additionally, TO in the oral and maxillofacial region are firm to palpation, whereas vascular anomalies are soft. Hence, this additional clinical feature may assist in differentiating between these two lesions.

TO development may result from one or more components of Virchow’s triad, namely, blood hypercoagulability, vascular wall injury, and altered blood flow.^4-6^ In the present multicenter study, 50 (26.7%) cases were associated with other lesions. Alteration in normal blood flow can lead to platelet aggregation, even in the absence of hypercoagulability or vascular wall injury.^4-6^ This mechanism may explain the thrombus formation observed in cases of hemangioma, papillary endothelial hyperplasia, and aneurysmal bone cyst. In addition, in oral varix, blood reflux and valve dysfunction can cause vascular wall damage.^25^ Conversely, in cases associated with actinic cheilitis and sialolith, the local inflammatory process and trauma may predispose to TO development.^6,26,27^ Moreover, chronic sun exposure induces alterations in the extracellular matrix and degradation of collagen fiber, which can also lead to vascular wall changes and promote thrombus formation.^28^

This study analyzed a large number of TO cases and contributed to defining the clinicopathological profile of patients affected by oral and maxillofacial thrombi, including their associated lesions. However, the main limitation of this study was the absence of complete clinical information, given its retrospective nature and reliance on biopsy records. This limitation likely stems from differences in data collection protocols used during clinical care. Bearing this in mind, it is essential that anatomopathological request forms be completed accurately. Another limitation was the lack of information about systemic etiological factors, such as the use of oral contraceptives, the occurrence of TO in other anatomical regions, and arterial hypertension. It should also be emphasized that the clinical information provided to pathology services should not be limited to the standard fields of the biopsy request form. Despite the benign nature of these lesions, a comprehensive evaluation of the patient’s systemic condition is essential, and effective communication among health professionals must be maintained, since the relationship between oral and maxillofacial thrombi and systemic factors is still uncertain.

## Conclusion

In summary, TOs may be underdiagnosed in oral and maxillofacial biopsy samples. Our multicenter study revealed a higher frequency of TOs among females aged 60–69 years. The lip was the most commonly affected anatomical site, and the most frequent morphological type was the organizing thrombus. In addition, TOs were frequently associated with oral varices. This study provides clinicians with valuable information that may assist in diagnosing oral and maxillofacial thrombi.

## Data Availability

The data supporting the findings of this study are available from the corresponding author upon reasonable request.

## References

[1] Furie B, Furie BC (2008). Mechanisms of thrombus formation. N Engl J Med.

[2] Lippi G, Favaloro EJ, Franchini M, Guidi GC (2009). Milestones and perspectives in coagulation and
hemostasis. Semin Thromb Hemost.

[3] Barbosa ACS, Palma DI, Melo MK, Costa AK, França GM (2024). Cross-sectional study of sublingual varicosities: systemic
exposures. Oral Maxillofac Surg.

[4] Esmon CT (2009). Basic mechanisms and pathogenesis of venous
thrombosis. Blood Rev.

[5] Lijfering WM, Rosendaal FR, Cannegieter SC (2010). Risk factors for venous thrombosis - current understanding from
an epidemiological point of view. Br J Haematol.

[6] Previtali E, Bucciarelli P, Passamonti SM, Martinelli I (2011). Risk factors for venous and arterial thrombosis. Blood Transfus.

[7] Wendelboe AM, Raskob GE (2016). Global burden of thrombosis: epidemiologic
aspects. Circ Res.

[8] Heit JA (2015). Epidemiology of venous thromboembolism. Nat Rev Cardiol.

[9] Raskob GE, Angchaisuksiri P, Blanco AN, Buller H, Gallus A, Hunt BJ (2014). Thrombosis: a major contributor to the global disease
burden. J Thromb Haemost.

[10] Shatzel JJ, O'Donnell M, Olson SR, Kearney MR, Daughety MM, Hum J (2019). Venous thrombosis in unusual sites: a practical review for the
hematologist. Eur J Haematol.

[11] Tjioe KC, Oliveira DT, Santos PS (2015). Tongue phlebothrombosis: pathogenesis and potential
risks. Quintessence Int.

[12] Lima GMG, Moraes RM, Cavalcante AS, Carvalho YR, Anbinder AL (2015). An isolated phlebolith on the lip: an unusual case and review of
the literature. Case Rep Pathol.

[13] Milhan NVM, Torquato LC, Costa V, De Marco AC, Carvalho YR, Anbinde AL (2018). A mixed form of intravascular papillary endothelial hyperplasia
in an uncommon location: case and literature review. Dermatol Online J.

[14] Davila-Villa P, Padilla-Rosas M, Meza-García G, Nava-Villalba M (2022). Vascular malformation of tongue with phlebothrombosis/phlebolith
in a young patient: an unusual presentation. BMJ Case Rep.

[15] Cankaya H, Unal O, Ugras S, Yuca K, Kiris M (2003). Hemangioma with phleboliths in the sublingual gland: as a cause
of submental opacity. Tohoku J Exp Med.

[16] Matsumura Y, Inui M, Nomura J, Yanase S, Nagai K, Tagawa T (2004). A case of thrombosis mimicking a buccal tumour: usefulness of
MRI. Dentomaxillofac Radiol.

[17] Elm E, Altman DG, Egger M, Pocock SJ, Gøtzsche PC, Vandenbroucke JP (2014). The strengthening the reporting of observational studies in
epidemiology (STROBE) Statement: guidelines for reporting observational
studies. Int J Surg.

[18] Jolugbo P, Ariëns RA (2021). Thrombus composition and efficacy of thrombolysis and
thrombectomy in acute ischemic stroke. Stroke.

[19] Wang C, Hang Y, Cao Y, Zhao L, Jiao J, Li M (2023). A nomogram for predicting thrombus composition in stroke patients
with large vessel occlusion: combination of thrombus density and
perviousness with clinical features. Neuroradiology.

[20] Tobouti PL, Pigatti FM, Martins-Mussi MC, Machado de Sousa SC (2017). Oral Thrombus: report of 122 cases with clinically descriptive
data. Med Oral Patol Oral Cir Bucal.

[21] Lowe GD (2008). Common risk factors for both arterial and venous
thrombosis. Br J Haematol.

[22] Sato H, Takeda Y, Satoh M (2002). Expression of the endothelial receptor tyrosine kinase Tie2 in
lobular capillary hemangioma of the oral mucosa: an immunohistochemical
study. J Oral Pathol Med.

[23] Buckmiller LM, Richter GT, Suen JY (2010). Diagnosis and management of hemangiomas and vascular
malformations of the head and neck. Oral Dis.

[24] Flucke U, Karanian M, Broek RW, Thway K (2020). Soft tissue special issue: perivascular and vascular tumors of
the head and neck. Head Neck Pathol.

[25] Raffetto JD, Khalil RA (2008). Mechanisms of varicose vein formation: valve dysfunction and wall
dilation. Phlebology.

[26] Silva LV, Arruda JA, Abreu LG, Ferreira RC, Silva LP, Pelissari C (2020). Demographic and clinicopathologic features of actinic cheilitis
and lip squamous cell carcinoma: a Brazilian multicentre
study. Head Neck Pathol.

[27] Stark K, Massberg S (2021). Interplay between inflammation and thrombosis in cardiovascular
pathology. Nat Rev Cardiol.

[28] Rojas IG, Martínez A, Pineda A, Spencer ML, Jiménez M, Rudolph MI (2004). Increased mast cell density and protease content in actinic
cheilitis. J Oral Pathol Med.

